# Antimicrobial Membranes of Bio-Based PA 11 and HNTs Filled with Lysozyme Obtained by an Electrospinning Process

**DOI:** 10.3390/nano8030139

**Published:** 2018-03-01

**Authors:** Valeria Bugatti, Luigi Vertuccio, Gianluca Viscusi, Giuliana Gorrasi

**Affiliations:** Department of Industrial Engineering, University of Salerno, via Giovanni Paolo II, 132, 84084 Fisciano (SA), Italy; vbugatti@unisa.it (V.B.); lvertuccio@unisa.it (L.V.); gviscusi@unisa.it (G.V.)

**Keywords:** food packaging, electrospun fibers, nanoencapsulation, controlled release

## Abstract

Bio-based membranes were obtained using Polyamide 11 (PA11) from renewable sources and a nano-hybrid composed of halloysite nanotubes (HNTs) filled with lysozyme (50 wt % of lysozyme), as a natural antimicrobial molecule. Composites were prepared using an electrospinning process, varying the nano-hybrid loading (i.e., 1.0, 2.5, 5.0 wt %). The morphology of the membranes was investigated through SEM analysis and there was found to be a narrow average fiber diameter (0.3–0.5 μm). The mechanical properties were analyzed and correlated to the nano-hybrid content. Controlled release of lysozyme was followed using UV spectrophotometry and the release kinetics were found to be dependent on HNTs–lysozyme loading. The experimental results were analyzed by a modified Gallagher–Corrigan model. The application of the produced membranes, as bio-based pads, for extending the shelf life of chicken slices has been tested and evaluated.

## 1. Introduction

The possibility to extend the shelf life of packaged food is a goal that covers several areas of basic and applied research, such as chemistry, microbiology, and materials science. The critical need in the food packaging field is the application of effective methods to inactivate spoilage and foodborne pathogens on the surface of food products [1]. Antimicrobial agents, directly incorporated into food packaging materials, are able to extend the shelf life of packaged foods, thus sustaining its nutritional and sensory qualities [2]. The antimicrobial agents used in food packaging materials generally include inorganic, organic, and biological active molecules. The antimicrobials classified as natural, efficient, and non-toxic are preferred due to the health and ecological concerns. Nanoscale antimicrobial materials attracted much attention due to their improved antimicrobial activities compared with traditional packaging. There are several technological approaches for preparing nanomaterials, and among these the electrospinning process is one of the most attractive methods due to its continuous fabricating capability and simple operating process [3,4]. The obtained materials possess high surface area to volume ratio making it suitable for various applications, such as filters [5,6], absorbing materials [7], textiles [8], sensors [9] and scaffolds for tissue engineering [10,11,12,13]. However, the application of electrospinning in the field of food packaging is still less explored [14,15,16]. Polyamide-11 (PA 11) is a 100% bio-renewable material. It is a high-performance, semi-crystalline, thermoplastic polymer entirely derived from castor oil. When compared to petroleum-based nylons and other conventional plastics, PA 11 has low net CO_2_ emissions and global warming potential. Some of the outstanding properties of PA 11 include high impact and abrasion resistance, low specific gravity, excellent chemical resistance, low water absorption, high thermal stability, and capability to be processed over a wide range of temperatures. PA 11 has also excellent dimensional stability, and maintains physical properties over a wide range of temperatures and environments. The possibility to add small amount of nanofillers into polymer matrices is a valuable strategy to obtain novel materials with new properties and added functionalities. Very recently PA 11 has been mixed with clay-based fillers [17,18,19,20,21,22,23], carbon-based nanomaterials [24,25,26,27,28,29], and inorganic particles [30,31] in order to improve its thermal, mechanical, rheological, and electrical properties. In the last few years a new class of natural clays are attracting great interest as fillers for polymers, the halloysite nanotubes (HNTs). They are green materials, cheap, and available in thousands of tons from natural deposits. HNTs have an average length of about 1000 nm, an internal diameter (lumen) of about 10–15 nm, and external diameter of about 50–80 nm. Their general chemical formula is Al_2_Si_2_O_5_(OH)_4_ × nH_2_O, with a predominant form of hollow tubes, similar to kaolin structure, but with the alumosilicate sheets rolled into tubes [32,33,34,35]. The HNTs external surface is composed of Si–O–Si groups, whereas the internal surface consists of a gibbsite-like array of Al–OH groups. HNTs can be dispersed in polymeric matrices without exfoliation, as required for a good dispersion of layered clays, due to the tubular shape and less abundant –OH groups on the surface. Polymeric materials have been filled with these tubular nano-containers [36,37,38,39] for the release of specific active molecules (antimicrobial, drugs, essential oils, flame retardant, self-healing, anticorrosion, etc.) in specific environments [40,41,42,43,44,45,46]. Very recently, halloysite nanotubes were also used for enzyme immobilization to study the enzymatic activity [47,48,49,50]. The present study aims to report the formulation and preparation of bio-based composite membranes based on halloysite as nanocontainers for lysozyme, as natural antimicrobial agent, and PA 11. Lysozyme is a natural antimicrobial agent categorized as GRAS (Generally Recognized as Safe) by the U.S. Food and Drug Administration (FDA), indicating it can be used in food industry without further approval. The nano-hybrid loading into the electrospun solutions was 1.0%, 2.5%, and 5.0%. The technique used for the membrane preparation was electrospinning. The morphology and structure of the membranes were investigated and correlated to the nano-filler loading and the sustained release of lysozyme molecules. Application of the produced membranes as bio-based pads for extending the shelf life of chicken slices has been tested and evaluated.

## 2. Experimental

### 2.1. Materials

PA11 with *ρ* = 1.026 g·cm^−3^ at *T* = 25 °C, glass transition temperature *T_g_* = 46 °C and melting temperature *T_m_* = 198 °C (CAS 25035-04-5), halloysite nanoclay powders (CAS 1332-58-7), lysozyme powders (CAS 12650-883), hexafluoroisopropanol (HFiP) (CAS 920-66-1). All materials were supplied from Sigma Aldrich (Milan, Italy) and used as received. The preparation of the nano-hybrid HNTs–lysozyme was carried out accordingly to a previously reported procedure [51]. 3 g of lysozyme were dissolved in 30 mL of water at 50 °C for 20 min. The HNTs (3 g) were then added to the lysozyme solution. Ultrasonic processing was performed for 10 min to make HNTs sufficiently dispersed in the lysozyme solution. Vacuum (0.085 MPa) was applied to remove the air between and within the hollow tubules for 15 min. The solution was then taken out from the vacuum and shaken for 5 min. Vacuum was re-applied for 15 min, to remove the trapped air. The HNTs loaded with lysozyme were dried in an oven for 16 h at 50 °C to reach a constant weight. The procedure above described was repeated twice. The content of lysozyme (wt %) in the HNTs–lysozyme hybrid, using the TGA analysis, was estimated to be around 50 wt %. The lysozyme content much exceed the loading capacity of the halloysite nanotubes. This detected amount is then relative either to the molecules inside the nanotubes, or to the molecules external to the nanotubes, that concur with different modes to the release (see Section 3, discussion on controlled release of lysozyme).

### 2.2. Electrospinning Procedure

The solutions for electrospinning were prepared using the following ratio between the different components: 0.3 g of PA11 were added to 2.7 g of HFiP (sample pure PA11); 0.297 of PA11 and 0.003 g of HNTs–lysozyme were added to 2.7 g of HFiP (sample PA11/1% HNTs–lysozyme); 0.292 of PA11 and 0.0075 g of HNTs–lysozyme were added to 2.7 g of HFiP (sample PA11/2.5% HNTs–lysozyme); 0.285 of PA11 and 0.015 g of HNTs–lysozyme were added to 2.7 g of HFiP (sample PA11/5% HNTs–lysozyme). The mixed solutions were placed into a 5 mL plastic syringe. An electrode lead of a high voltage power supply (HV Power Supply, Gamma High Voltage Research, Ormond, FL, USA) was connected to the needle tip (internal diameter 0.84 mm) of the syringe. A constant positive DC voltage potential was fixed at 16 kV. A syringe pump (NE-1000 Programmable Single Syringe Pump, New Era Pump Systems Inc., Farmingdale, NY, USA) was used to feed the needle with polymer solution at volumetric flow rate of 1 mL/h. For collecting of the fibrous mats, aluminum plates of 10 × 10 mm^2^ were placed on a grounded aluminum collector, the distance between the collector and the syringe was 20 cm. The solutions were subjected to electrospinning in a closed chamber where the temperature was controlled at 24 °C and the relative humidity at 35%. Table 1 reports the samples and the average fibers diameters.

### 2.3. Methods of Analysis

SEM analysis on the electrospun samples was performed with a LEO 1525 microscope. The fiber diameters were detected, in the SEM images, through one-by-one localization, using the software Sigma SCAN (Analyze Images Automatically) considering 500 fibers for every system (Systat Software Inc., San Jose, CA, USA). The use of the software “OriginLab” (Systat Software Inc., San Jose, CA, USA) allowed the evaluation of distribution of diameters for all systems.

Fourier transform infrared (FT-IR) were recorded using a Bruker spectrometer, model Vertex 70 (Bruker Italia, Milano, Italy) (average of 32 scans, at a resolution of 4 cm^−1^).

The mechanical propertie*s* of the samples were evaluated from stress–strain curves obtained using a dynamometric apparatus INSTRON 4301 (ITW Test and Measurement Italia S.r.l., Pianezza, Italy). The experiments were conducted at room temperature with the deformation rate of 5 mm/min. The initial length of the samples was about 10 mm. Elastic moduli were derived from the linear part of the stress–strain curves, giving the sample a deformation of 0.1%. Reported results are the average of data obtained from five samples.

The release kinetics of the lysozyme were performed by ultraviolet spectrometric measurement using a Spectrometer UV-2401 PC Shimadzu (Shimadzu, Kyoto, Japan). The tests were performed using rectangular specimens of 4 cm^2^ and the same thickness (150 μm), placed into 25 mL physiological solution and stirred at 100 rpm in an orbital shaker (VDRL MOD. 711+) (ASAL S.R.L., Milan, Italy). The release medium was withdrawn at fixed time intervals and replenished with fresh medium. The considered band was at 265 nm.

Antimicrobial analyses were performed considering 1 g of chicken meat, stored at 4 °C, collected at storage times of 6, 9, and 13 days and added to 9 mL of saline peptone water. The mixture was homogenized for 1 min in a stomacher 400 (Lab Blender, Seward Medical, London, UK) and 1 mL of homogenate subjected to serial dilutions in the same diluent. Aliquots of 0.1 mL of different dilutions were spread onto the culture media selective for *Pseudomonas* (Oxoid, Rodano, Italy). Microorganisms were enumerated with the method based on count of Colony Forming Units (CFU), by using 25–250 CFU plates as range of countable colonies. *Pseudomonas* spp. were selected on *Pseudomonas* (strain PAO1) agar base supplemented with (with selective supplement, CFC) at 30 °C for 72 h (ISO/TS 11059:2009 (IDF/RM 225:2009)) (Oxoid, Rodano, Italy). Chicken breasts were analyzed at 24 h (*t* = 0) postmortem and were cut in small fillets of about 3 × 1 × 1 cm, weight ≅ 5–6 g, put on the pads made of unfilled PA11 and PA11 + 5 wt % of HNTs/lysozyme. Samples were stored at 4 °C and examined at intervals of 6, 9, and 13 days. Three samples were analyzed. Reported results are the average of three replicates.

## 3. Results and Discussion

**Figure 1:**
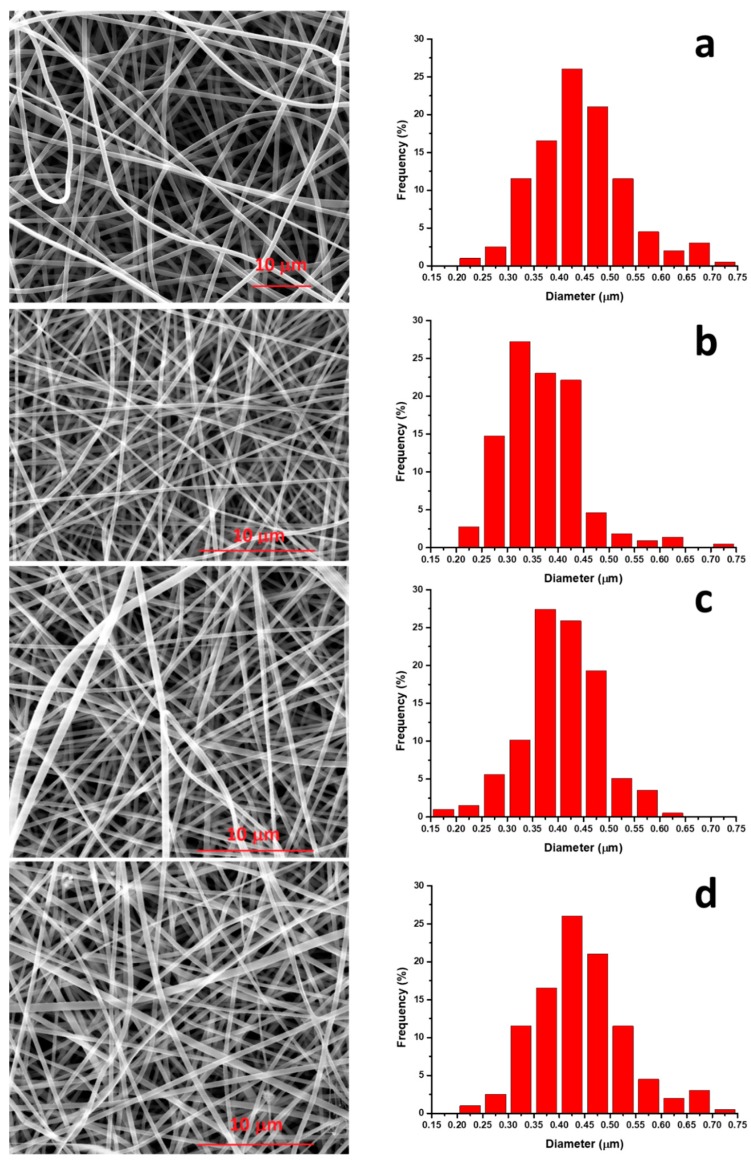
SEM pictures for samples (**a**) PA11, (**b**) PA11/1.0 wt % HNTs–lysozyme, (**c**) PA11/2.5 wt % HNTs–lysozyme, (**d**) PA11/5.0 wt % HNTs–lysozyme.

Figure 1 reports the SEM micrographs of unfilled PA 11 and its composites with various filler loading. It is evident that the electrospinning process, in the experimental condition described in Section 2.2, led in all cases to the formation of randomly oriented, defect-free cylindrical fibers, with a very narrow average fiber diameter (~0.3–0.5 μm).

**Figure 2:**
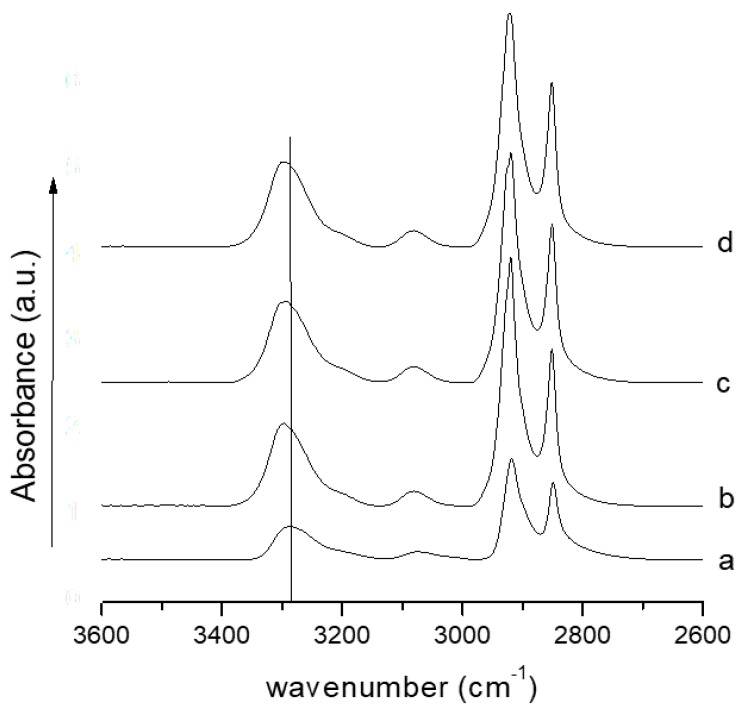
FTIR spectra for samples (a) PA11, (b) PA11/1.0 wt % HNTs–lysozyme, (c) PA11/2.5 wt % HNTs–lysozyme, (d) PA11/5.0 wt % HNTs–lysozyme.

Figure 2 reports the FTIR spectra of membranes of PA11 and composites in the wavenumber range 2600–3600 cm^−1^. It can be observed that the band relative to the N–H stretching amide I (3283 cm^−1^) of the polymer [52] results shifted to higher wavenumber (3297 cm^−1^) for all the composites. This can be due to hydrogen bonds between the nitrogen of the amidic group of the PA11 and the hydrogen of the carboxyl group of lysozyme molecules external to the HNTs and/or free dispersed into the polymer matrix.

**Figure 3:**
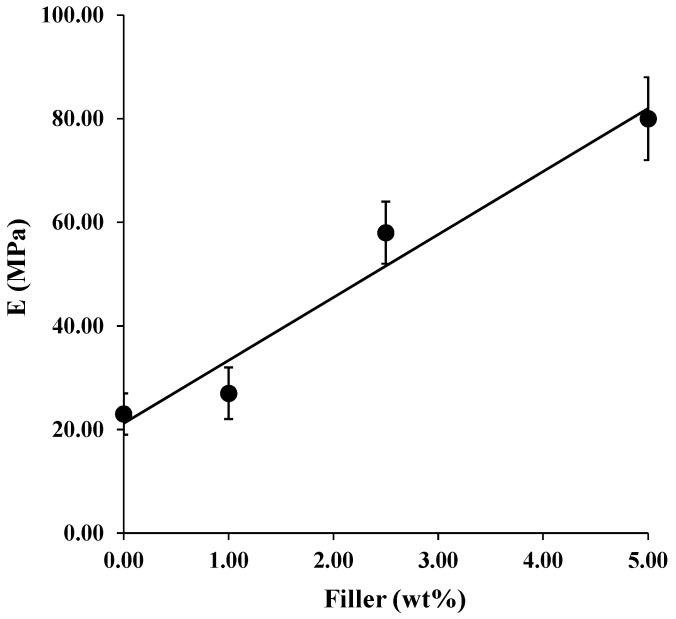
Elastic modulus, E (MPa), as a function of filler content.

The mechanical properties, in terms of elastic modulus E (MPa), are reported in Figure 3 as a function of filler loading. In the case of composite systems, the enhancement of the mechanical properties of nanocomposites requires a high degree of load transfer between the continuous and dispersed phases. If the interfacial adhesion between the phases is weak, the filler behaves as holes or nanostructured flaws, introducing local stress concentrations, and its benefits on the properties are lost. The filler must be well dispersed, because in the case of poor dispersion, the strength is significantly reduced. The modulus linearly increases with HNTs–lysozyme in all the investigated composition range. The improvement of this parameter can be attributed to the presence of HNTs that act as reinforcing agents for the polymer matrix, well dispersed into the polymer at any composition. Data are reported in Table 1.

**Figure 4:**
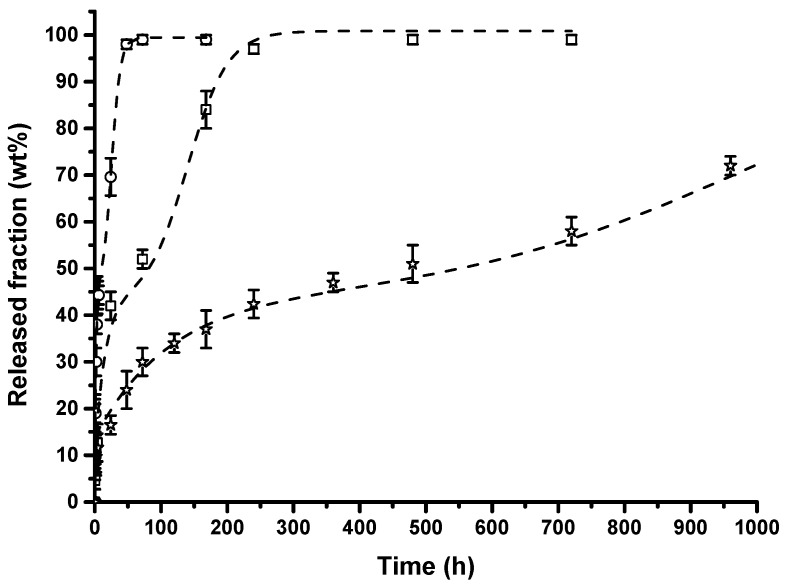
Released fraction (wt %) as a function of time (hour) for samples: (◯) PA11/1.0 wt % HNTs–lysozyme, (☐) PA11/2.5 wt % HNTs–lysozyme, (☆) PA11/5.0 wt % HNTs–lysozyme.

Figure 4 reports the release fraction of lysozyme (wt %) versus time (hours) for the considered composite membranes. The release mode for each sample is very complex and a composition of several de-intercalation processes. It is possible to visualize the entire release in three main steps. The initial one is related to the burst and is due to the free lysozyme molecules that are external to the nanofibers and external to the HNTs outside the nanofibers and present on the membranes’ surface. The burst’s entity increases with the increasing of filler loading in the composites. A second step of release can be attributed to the diffusion of the lysozyme molecules from the bulk of the PA11 nanofibers and external to the HNTs. In the third step, the release mode can be interpreted as desorption and diffusion of lysozyme from the inside of the halloysite nanotubes. It is interesting to note that the total amount of lysozyme released decreases with the filler content, at the same time, and in the investigated range of time (41 days), the release does not reach 100% for the membrane filled with 5 wt % of HNTs–lysozyme (i.e., 2.5% of lysozyme). An empirical equation was adopted, based on a modified Gallagher–Corrigan model [53], that has already been used to describe the lysozyme release from nano-hybrid composite films [46,51]. This model (Equation (1)) presents the combination of two consecutive kinetic mechanisms (dual-model diffusion model). A constant parameter (A) was introduced to take into account the initial burst release. The addition of this parameter simply shifts the model predictions vertically. If no burst occurs, A is zero and the equation is reduced to the original equation.

(1) $$X\left(t\right)=A+X_{1}\left(1-e^{-C_{1}t}\right)+X_{2}\left(\frac{e^{-C_{2}\left(t_{max}-t\right)}}{\left(1+e^{-C_{2}\left(t_{max}-t\right)}\right)}\right)$$

In Equation (1), *X*(*t*) is the fraction of lysozyme released at the time t; *X*_1_ (%) and *X*_2_ (%) are the relative amounts of lysozyme released in the first and second steps of the mechanism; *C*_1_ and *C*_2_ are the kinetic constants of the first and second steps of the release mechanism, *t*_max_ is time characteristic of the second step mechanism, and *A* is the burst parameter. Figure 4 also reports the model results for the three analyzed systems as dotted lines. The experimental results for the three considered membranes are well fitted by the model. It is a further confirmation that, apart the initial burst, two distinct steps can occur in the release mechanism. Table 2 reports the model parameters obtained by a nonlinear least squares fitting procedure. Both the rate constant (*C*_1_) and the amount of lysozyme released (*X*_1_) in the initial stage of release slightly decreased with the filler content. It is hypothesized that such behavior is related to the hindrance effect created by the increasing percentage of halloysite nanotubes. The increase in the filler content also generates a delay in the second step time (*t*_max_) with a diminution in the resulting rate constant (*C*_2_). 

In order to test the antimicrobial activity against *Pseudomonas*, we used the prepared composites at 5 wt % of HNTs–lysozyme as pads for chicken meat. *Pseudomonas* spp. are specific microbial species which are representative of the microbial dynamics during meat spoilage [54,55]. They are grouped into psychrotrophic microbial groups [54], commonly linked with fresh meat spoilage and with the ability to grow during storage in air, vacuum, and modified atmosphere packaging. Consequently, they are meat colonizers and an important portion of the spoilage microbiota, being occasionally the dominant organisms.

**Figure 5:**
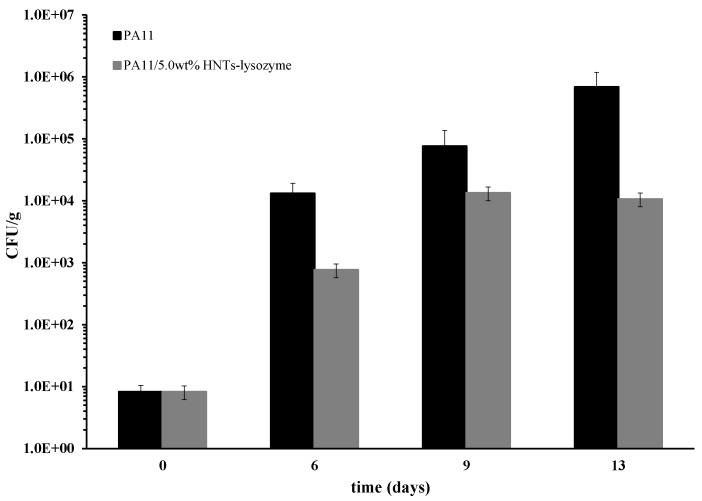
CFU/g of *Pseudomonas* on chicken slices, evaluated at 4 °C, comparing samples PA11 and PA11/5.0 wt % HNTs–lysozyme, as function of storage time.

To simulate the meat storage in the field of large food distribution, we put the prepared membranes as pads inside petri dishes of 4 cm diameter. The filled petri dishes were covered with food grade cellophane film. Figure 5 reports the values of CFU/g of *Pseudomonas* for samples evaluated at 6, 9, and 13 days of storage at 4 °C. A pad made of unfilled PA 11 was used as reference. The starting bacterial count is quite low and after six days, had increased by four orders of magnitude for the membrane pad of PA11 and two orders of magnitude for the pad made of composite filled with 5 wt % of HNTs–lysozyme. The bacterial count increases with the time, being always lower for the sample put on the composite material. It is interesting to note that a plateau value is reached for the sample PA11+ 5 wt % HNTs–lysozyme, one order of magnitude lower than the PA11 at 13 days.

## 4. Concluding Remarks

In this paper, the preparation of bio-based membranes composed of Polyamide 11 (PA11) from renewable sources and lysozyme encapsulated into halloysite nanotubes (HNTs) is reported. The lysozyme content of the HNTs–lysozyme hybrid was ≅50 wt %. The hybrid loading into the PA11 was 1.0, 2.5, 5.0 wt %.

- SEM analysis revealed that, with the used processing conditions, both PA11 membrane and the composites show a narrow average fiber diameter (0.3–0.5 μm).
- The FTIR analysis revealed a shift of the N–H stretching of amide I, indicative of a good interaction between the PA11 and lysozyme molecules.
- The mechanical properties, in terms of elastic modulus, increase with filler content for the reinforcing effect of the HNTs.
- The release kinetics of composites’ membranes were found to be dependent on the nano-hybrid loading and were well fitted with a modified Gallagher–Corrigan model. It was demonstrated that varying the filler loading it is possible to tune the lysozyme release for desired applications.
- The membranes were used as antimicrobial pads for chicken meat storage. The membrane filled with 5.0 wt % of HNTs–lysozyme was tested against *Pseudomonas* growth for up to 13 days and compared with the unfilled PA11. A reduction in bacterial growth was found for the membrane filled with the antimicrobial compound.

## Figures and Tables

**Table 1:** Values of elastic modulus (MPa) for all samples (data plotted in Figure 3).

Filler Loading (wt %)	E (MPa)
0	23 ± 8
1.0	27 ± 16
2.5	58 ± 12
5.0	80 ± 18

**Table 2:** Values of the release model parameters evaluated using Equation (1).

Sample	*A*	*X*_1_ (%)	*X*_2_ (%)	*t*_max_ (h)	*C*_1_ (h^−1^)	*C*_2_ (h^−1^)	R^2^
PA11/1.0 wt % HNTs–lysozyme	6	39	55	25	4.91 × 10^−1^	1.46 × 10^−1^	0.985
PA11/2.5 wt % HNTs–lysozyme	9	34	58	140	6.57 × 10^−2^	3.30 × 10^−2^	0.987
PA11/5.0 wt % HNTs–lysozyme	13	30	57	984	9.07 × 10^−3^	4.37 × 10^−3^	0.985
